# Role of pretty nanoflowers as novel versatile analytical tools for sensing in biomedical and bioanalytical applications

**DOI:** 10.1002/SMMD.20230040

**Published:** 2024-02-26

**Authors:** Seyma Dadi, Ismail Ocsoy

**Affiliations:** ^1^ Department of Nanotechnology Engineering Abdullah Gül University Kayseri Turkey; ^2^ Department of Analytical Chemistry Faculty of Pharmacy Erciyes University Kayseri Turkey

**Keywords:** analytical applications, biosensor, catalytic activity, organic inorganic hybrid nanoflowers

## Abstract

In recent years, an encouraging breakthrough in the synthesis of immobilized enzymes in flower‐shaped called “organic‐inorganic hybrid nanoflowers (hNFs)” with greatly enhanced catalytic activity and stability were reported. Although, these hNFs were discovered by accident, the enzymes exhibited highly enhanced catalytic activities and stabilities in the hNFs compared with the free and conventionally immobilized enzymes. Herein, we rationally utilized the catalytic activity of the hNFs for analytical applications. In this comprehensive review, we covered the design and use of the hNFs as novel versatile sensors for electrochemical, colorimetric/optical and immunosensors‐based detection strategies in analytical perspective.

## Graphical abstract

Formation of nanoflowers and their biosensor function in biomedical and bioanalytical applications.

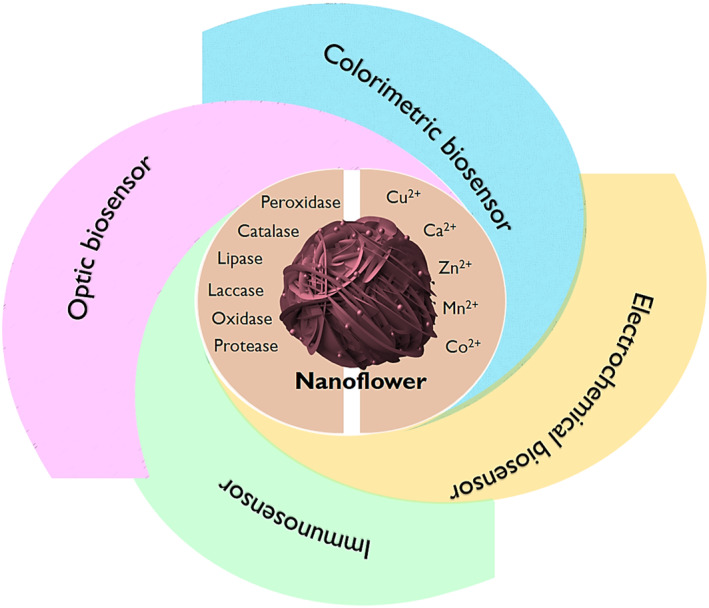

**Key points**

- The enzyme‐inorganic hybrid nanoflowers (hNFs) have shown higher catalytic activity compared to free enzymes.
- The hNFs were designed as a facile analytical tool for sensing different analytes.
- The biosensor applications of hNFs based on their catalytic activities are discussed.

## 1 INTRODUCTION

Enzymes are highly active biomolecules that can catalyze the reactions in different systems.^1^ They have wide applications in various scientific and industrial fields including drug synthesis, dye removal and biosensing due to their high catalytic activity, specificity, water solubility and low toxicity.^2^ However, free enzymes lost most of their initial activities at strong acidic and basic pH values (pHs) under room temperatures (RT = 25°C), and in the presence of organic solvents owing to easy degradation, then which hampered their application. In addition to that, they cannot be separated from reaction conditions for reuse.^3^, ^4^, ^5^, ^6^ Conventional enzyme immobilization techniques including adsorption, entrapment, covalent binding and cross‐linking were considered as a remedy to overcome these issues.^7^, ^8^ While immobilized enzymes exhibited quite high stability and even reusability, they lost most of their activities compared to the free form due to unfavorable conformation or the inhibiting of the active site of enzymes, which induces certain mass transfer limitations between the enzyme and substrate.^9^, ^10^, ^11^, ^12^ For instance, various materials such as carbon nanotubes,^13^ graphene oxide (GO)^14^ or natural polymers^15^ have been used as supports for enzyme immobilization, and a significant decrease in catalytic activities were recorded. Therefore, there is an indispensable demand to develop a new generation immobilization approach that may result in an increase in both catalytic activity and stability of enzymes. Up to date, several different flower‐shaped metallic nanomaterials (NMs) have been generated for a variety of applications, especially in analytical science.^16^, ^17^, ^18^ However, the flower‐shaped organic and inorganic hybrid NMs were discovered with an encouraging and breakthrough approach by Zare and co‐workers.^19^ These hybrid NMs called “Nanoflowers (NFs)” consisting of generally enzymes as organic component and copper (II) ions (Cu^2+^) as inorganic component have showed greatly enhanced catalytic activities and stabilities. The researchers recently have used non‐protein molecules as an alternative to enzymes to produce the NFs with intrinsic peroxidase‐like activities.^20^, ^21^, ^22^ Enzymes are still the most widely studied organic components for the production of the NFs with different metal ions.^23^, ^24^, ^25^, ^26^ Enzyme‐inorganic hybrid NFs (hNFs) have exhibited several fold increase in catalytic activities compared to free enzymes and conventionally immobilized enzymes owing to favorable enzyme conformation, porous structure and high surface area.^27^ In addition to those non‐enzyme molecules‐incorporated hNFs show remarkable peroxidase‐like activity in the presence of hydrogen peroxide (H_2_O_2_) relied on Fenton‐like reaction.^25^, ^28^, ^29^ Recently, the hNFs formed of either enzymes or non‐protein molecules has become a versatile analytical tool for sensing of various targets in the scope of analytical applications.^30^, ^31^, ^32^, ^33^ In this review, we have focused on preparation and use of the hNFs as biosensors in electrochemical, optical and colorimetric‐based detection of target. We have also emphasized the analytical applications of the hNFs benefiting from their catalytic activities.

## 2 SYNTHESIS OF hNFs

The hNFs, new generation hybrid NMs, were accidentally discovered by Zare and co‐workers in 2012.^19^ In a typical synthesis procedure, freshly prepared CuSO_4_ solution was added to phosphate buffered saline (PBS) solution (10 mM, pH 7.4) containing 0.1 mg mL^−1^ bovine serum albumin, then the mixture was incubated for 3 days at RT. After incubation, a blue precipitate appeared at the bottom of the mixture, which is considered as indication of hNFs.^19^ The self‐assembled hNFs is environmentally friendly due to the fact that it is easily synthesized in PBS at RT without any toxic chemicals.

The formation mechanism of hNFs is proposed with three steps including nucleation, growth and completion.^24^ At the nucleation step, metal ions react with negatively charged phosphate groups through electrostatic interaction to form metal‐phosphate crystals. These crystals bind to amine groups’ protein backbone via the coordination bonds. Thus, the reaction between metal‐phosphate crystals and amino groups provides protein‐metal‐phosphate primary nanocrystals. In the second step, these primary nanocrystals are fed with protein molecules to form separate nanopetals. These protein‐incorporated nanopetals bind to each other by acting as a glue function of protein. At the final step, the growth of the hNFs is saturated through the anisotropic growth to form complete hNFs.^34^, ^35^

The formation of hNFs is influenced by certain experimental parameters such as the concentration of organic and inorganic components, pHs, temperature and incubation time.^36^, ^37^, ^38^, ^39^, ^40^, ^41^, ^42^ The formation of hNFs depends on pHs of reaction environments which is in charge of the electrostatic interaction or between enzyme molecules and enzyme and metal ion.^36^ When the pH was below or above the isoelectric point (pI) of the enzyme, the formation of hNFs was hampered by the repulsion between positively or negatively charged enzyme molecules. For instance, Somturk et al. demonstrated how the pH of PBS influenced the morphology of urease‐Cu_3_(PO_4_)_2_ hNFs. The hNFs were formed at pH 6 (which is close to pI value of urease enzyme) because the enzyme is in the neutral form at that pH of 4. However, no hNFs formation was observed below 5 or above 9 owing to strong positive and negative repulsions, respectively, between enzyme molecules or between enzyme and Cu^2+^ ions.^37^

The incubation temperature is another important factor affecting the morphology of the hNFs. Wang et al. investigated the effect of the temperature on crystal growth and assembly of trypsin/Zn_3_(PO_4_)_2_ hNFs. While the particle size of the hNFs was not affected by the temperature, the thickness of the nanopetals of hNFs decreased from 150 to 100 nm as the temperature increased from 30 to 50°C. The reason is that high temperature triggered the rapid formation and assembly of nanopetals and reduced the thickness of the nanopetal layer.^38^, ^39^, ^40^

Although Cu^2+^ ion is widely used as an inorganic component in the synthesis of hNFs, different types of metal ions such as Ca^2+^, Co^2+^, Mn^2+^ can be used to obtain hNFs in different morphologies.^41^, ^42^, ^43^, ^44^ Escobar et al. investigated the effect of Ca^2+^, Cu^2+^ and Zn^2+^ ions on the morphology of *Thermomyces lanuginosus* Lipase hNFs (TLL hNFs). They showed that having different nucleation sites of TLL for metal ions affected the size and morphology of TLL hNFs. While Zn^2+^‐TLL hNFs has 2 μm compact and spherical shape and Cu^2+^ TLL hNFs had 1 μm spherical shape with rough surfaces, Ca^2+^ TLL hNFs presented 5–10 μm amorphous structures.^43^ In another study, Cu^2+^, Ca^2+^, Co^2+^ and Mn^2+^ were used as the metal ions to synthesize commercial protease “Neutrase” hNFs. Cu^2+^ and Co^2+^ hNFs presented the best ordered structure by assembling with well‐proportioned nanopetals resembling flowers like African marigold and rose, respectively. However, rather than a flower‐like appearance, Ca^2+^ and Mn^2+^ hNFs showed large amorphous aggregates. The reason for these results may be the difference in the arrangement of the outermost electron orbit of metal ions.^44^

The selection of enzyme plays a potent role in the synthesis of hNFs based upon their molecular weight and number of accessible NH_2_ groups in the protein structures.^45^, ^46^ The morphology and size of the hNFs were varied relied on enzyme type. For instance, Yu et al. synthesized hNFs using Ca_3_(PO_4_)_2_ as an inorganic component and six enzymes including bromelain, papain, trypsin, lipase from Porcine Pancreas, lipase from TLL and lipase B from *Candida antarctica* as organic component. The SEM images demonstrated that the morphology of the lipase hNFs was more like a flower than protease hNFs. The morphological diversity comes from the different coordination ability between Ca^2+^ ions and amide groups in the backbone of enzymes.^45^

## 3 APPLICATION OF hNFs

The hNFs have attracted considerable attention because of their superior catalytic activity and enhanced stability compared to free enzymes and traditional immobilized enzymes. The fact that hNFs have more active sites due to their large and porous surface area makes them ideal candidates for biosensing applications. In this section, we discuss the classification of biosensors based on various enzymes incorporated hNFs.

### 3.1 Electrochemical detection based on hNFs

**FIGURE 1:**
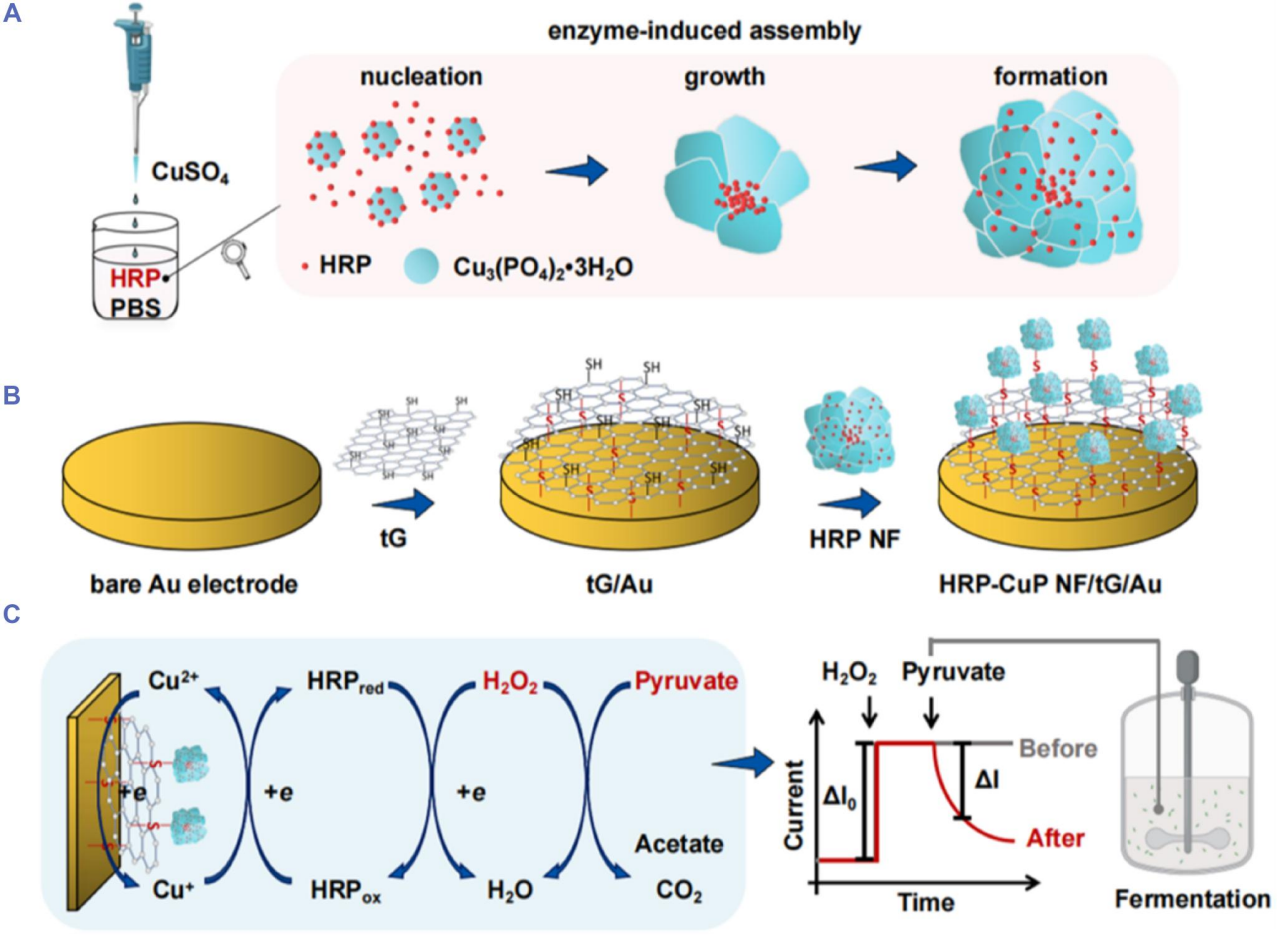
Schematic illustration of amperometric H_2_O_2_ and pyruvate detection mechanism. (A) Synthesis of HRP‐hNFs, (B) the self‐assembling of Au electrode with HRP‐hNFs and tG, (C) the mechanism of pyruvate detection strategy based on the consumption of H_2_O_2_ through the non‐enzymatic decarboxylation process of pyruvate. The performance of electrochemical biosensors can be improved by the incorporation of hNFs with other nanomaterials. Reproduced with permission.^50^ Copyright 2022, Elsevier. hNFs, hybrid nanoflowers.

Electrochemical biosensors are a class of biosensors which convert biochemical information related to the interaction of the analyte with the sensing element into electrical signals. Electrochemical biosensors are a good choice for the detection of biomolecules due to their excellent sensitivity, high accuracy, good selectivity, short detection time and affordability.^47^, ^48^ Recently, hNFs incorporated electrochemical biosensors have received a great deal of attention due to greatly improved catalytic activity and stability of the hNFs.^49^ For instance, Yang et al. reported a facile pyruvate determination method based on spontaneous decarboxylation reaction of pyruvate with H_2_O_2_ by using HRP hNFs instead of using pyruvate oxidase (POX). This was the first study to utilize H_2_O_2_ biosensor to determine the concentration of pyruvate in the microbial fermentation processes. General pyruvate detection is based on the oxidation of pyruvate in the presence of POX and the electrochemical detection of H_2_O_2_ produced by POX. As shown in Figure 1, in the proposed new detection procedure, the consumption of H_2_O_2_ by the non‐enzymatic decarboxylation of pyruvate causes to a signal decrease on the Au electrode immobilized with HRP hNFs and thiol graphene (tG). HRP‐hNFs/tG/Au electrode showed enhanced activity and conductivity owing to high catalytic activity and excellent electrochemical performance of the hNFs. Upon optimization, the developed H_2_O_2_ and pyruvate biosensor demonstrated a LOD of 0.06 and 0.3 mM (S/N = 3) in the linear range of 0.1–8.2 and 1–8 mM, respectively, as well as good accuracy and sensitivity.^50^

**FIGURE 2:**
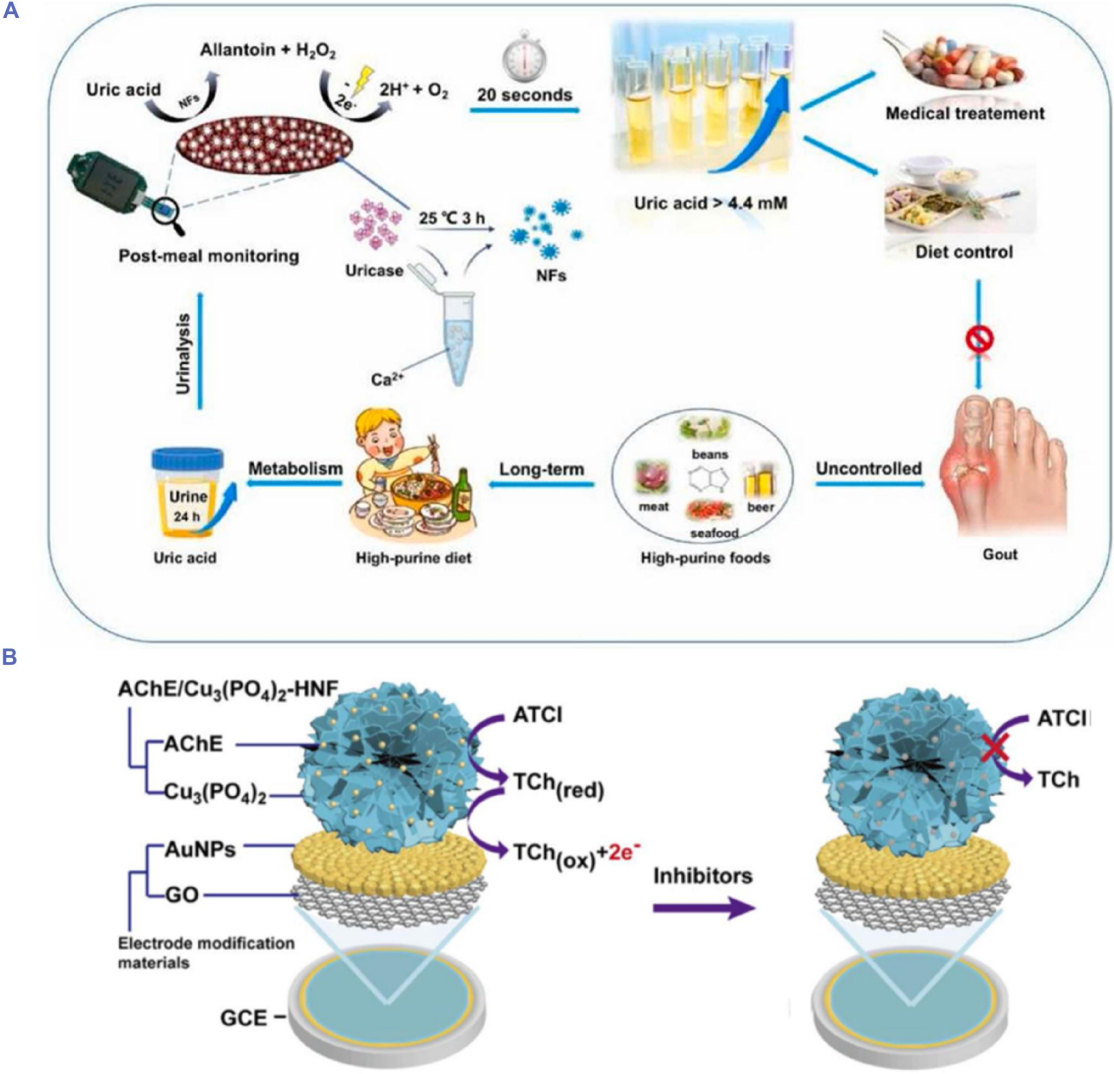
(A) The uricase‐hNFs‐based portable electrochemical uricometer for monitoring uric acid in urine samples. Reproduced with permission.^55^ Copyright 2022, Elsevier. (B) Schematic illustration of AChE based‐inhibition biosensor for the detection of OPs. Reproduced with permission.^56^ Copyright 2022, Royal Society of Chemistry. AChE, acetylcholinesterase; hNFs, hybrid nanoflowers; OPs, organophosphorous pesticides.

In a study conducted by Baek et al., GOx‐HRP hNFs‐Au NP‐decorated GO nanofibers (NFs) was fabricated for the electrochemical glucose biosensor. In this work, firstly, GO NFs were synthesized on the gold chip by the electrospinning method. Then, Au NPs were coated on the surface of GO NFs via electrostatic interaction and finally incorporated GOx‐HRP hNFs. The electrochemical performance of the biosensor was substantially enhanced due to the modification of the NFs with Au NPs, GO and GOx‐HRP hNFs, resulting in efficient electro‐catalyzed reaction. Accordingly, the obtained LOD was 0.018 μM with a wide linear range of 0.001–0.1 mM.^51^ In recent years, portable electrochemical sensors have been developed due to their cost effectiveness, easy use, short analysis time and simple sample preparation. Also portable electrochemical sensors have opened a new avenue of opportunities for monitoring human and animal health care, food and environmental contamination, etc.^52^, ^53^, ^54^ In a study by Xing et al., portable electrochemical uricometer (PUM) assisted with the uricase hNFs was reported due to the uricase hNFs possess better stability and good reusability (Figure 2A). Non‐invasive PUM was applied for determining the concentration of uric acid (UA) in urine samples at random intervals in 20 s. In this method, uricase hNFs on AuNPs‐screen printed carbon electrode catalyzed the oxidation of uricase to produce H_2_O_2_, which was measured by cyclic voltammetry or amperometry protocol. The designed PUM showed satisfying performance for determining uric acid with a low LOD of 0.82 μM and a wide linear range from 0 to 5 mM with non‐invasive analysis, repeat performance and interference ability. Furthermore, the developed PUM was also used to monitor and analyze the clinical urine samples from different volunteers. These results demonstrated that PUM had huge potential in practical home UA detection.^55^

Acetylcholinesterase (AChE) inhibition‐based biosensors are widely used for the analysis of organophosphorous pesticides (OPs) in vegetables, fruits and environmental samples. The catalytic activity of AChE was inhibited in the presence of trace amounts of OPs. In the general detection mechanism, acetylcholine is hydrolyzed by AChE to thiocholine, which is oxidized by applied voltage and generates a current signal. In the presence of OPs, AChE can be easily inhibited and causes a less production of thiocholine and decrease in current signal. In this way, the analysis of OPs can be achieved by measuring the decline of current.^57^, ^58^, ^59^ Yang et al. developed an electrochemical sensor system based on AChE hNFs for the detection of dichlorvos as model OPs. In the sensor system, Au NPs and GO were used to amplify the electrochemical signal and improve cell biosensing ability. AChE hNFs/Au NPs/GO/glassy carbon electrode showed favorable detection performance due to the AChE hNFs have large surface area and provide the affinity to substrates with improved catalytic activity (Figure 2B). This biosensor enabled the determination of dichlorvos with a detection limit of 0.07 pM and exhibited great detection accuracy and recoveries about 98.65%–103.43% in real agricultural samples.^56^ In another study, the same researchers reported the fabrication of in situ growth of aptamer (Apt) based AChE hNFs. They utilized the aptamer of AChE for the in situ growth of AptAChE hNFs on the carbon paper. The use of specific Apt of enzyme caused localisation of enzyme molecules on the electrode surface. The in situ growth enabled OPs to be analyzed with high sensitivity because it facilitated the exposure of catalytic activity and mass transfer between enzyme and substrate.^60^

### 3.2 Colorimetric/optical detection based on hNFs

Optical detection methods have been attractive and very popular for many years due to the sensitive and selective detection, rapidity of analysis, cost effectiveness and portability of instrumentation.^61^, ^62^ The colorimetric technique is classified under optical detection methods. Colorimetric sensors detect the color change in the presence of target molecules. The color change can be easily detected by the naked eye or instruments, including spectrophotometers, smartphone cameras or scanner devices.^61^, ^63^, ^64^, ^65^ Colorimetric and optical assays based on free enzymes suffer from some limitations such as low stability and recovery. Thus, hNFs assisted colorimetric and optical assays have been extensively used for sensitive and selective detection of different analytes. Herein, we have introduced some examples of hNFs‐based colorimetric and optical biosensors.

**FIGURE 3:**
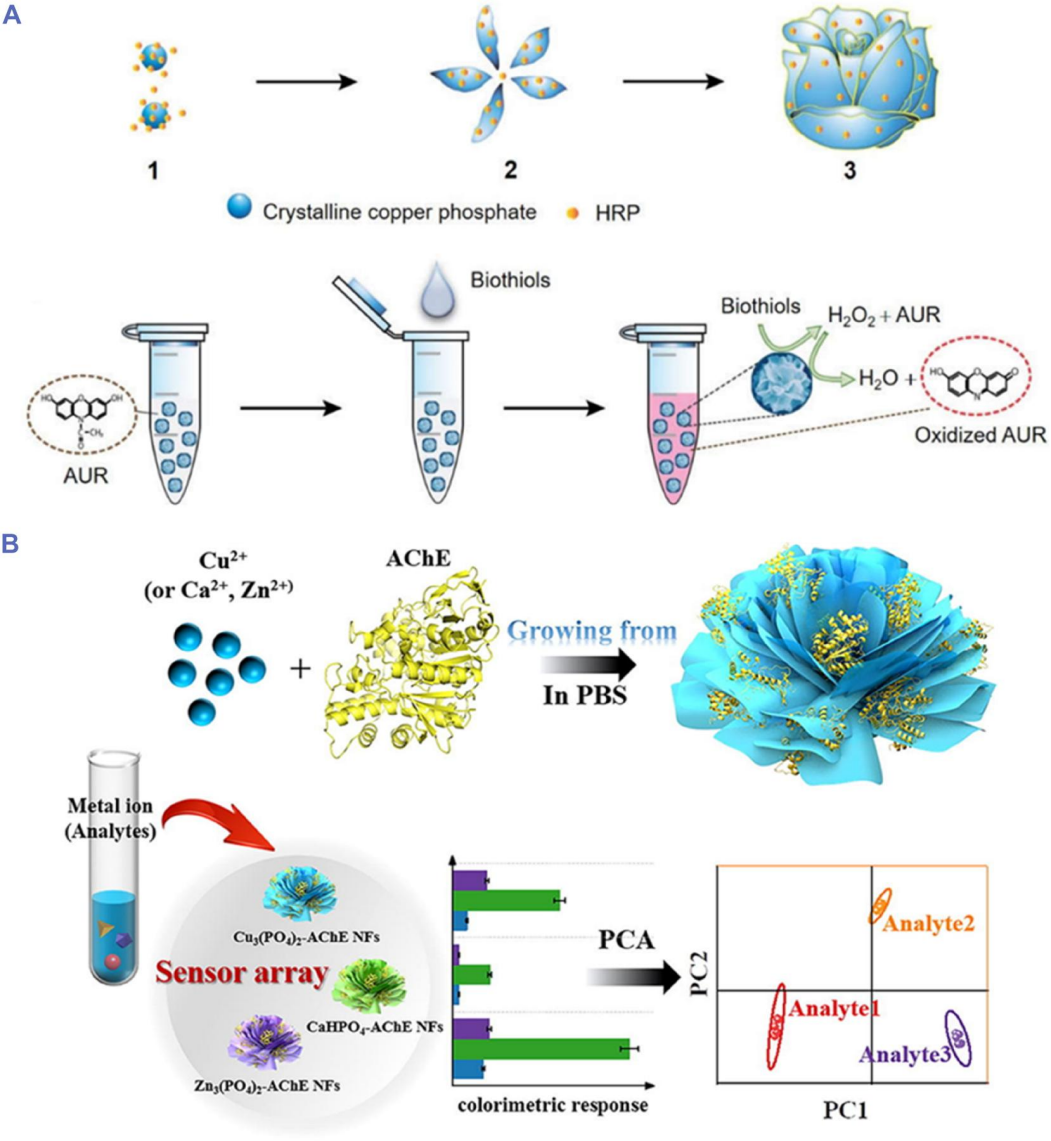
(A) Schematic illustration of synthesis of HRP‐hNFs and HRP‐hNFs based fluorescent one pot detection of biothiols. Reproduced under terms of the CC‐BY license.^66^ Copyright 2022, The Authors, published by MDPI. (B) schematic representation of the synthesis of AChE‐hNFs and the sensor array based on the AChE‐Cu_3_(PO_4_)_2_ hNFs, AChE‐CaHPO_4_ hNFs and AChE‐Zn_3_(PO_4_)_2_ hNFs for discrimination of metal ions with PCA. Reproduced with permission.^67^ Copyright 2022, Elsevier. AChE, acetylcholinesterase; hNFs, hybrid nanoflowers; PCA, principal component analysis.

Le et al. developed a fluorescent biosensor using HRP‐Cu_3_(PO_4_)_2_ hNFs for the detection of biological thiols, including glutathione (GSH), cysteine (Cys) and homocysteine (Hcy) (Figure 3A). For the first time, the researchers showed that Cu_3_(PO_4_)_2_ crystals had biothiol oxidase‐like activity to oxidase biothiols and thus, HRP‐Cu_3_(PO_4_)_2_ hNFs had dual enzymatic activities, which were applied to the cascade reaction system. HRP‐Cu_3_(PO_4_)_2_ hNFs catalyzed the biothiols and generate H_2_O_2_. Then these hNFs transformed the Amplex UltraRed (AUR) substrate to oxidized AUR (intense fluorescent product). The proposed method exhibited a detection limit of 13.4, 4.5, and 18.3 nM for GSH, Cys and Hcy, respectively, and showed high sensitivity in comparison with other reported biothiol sensors.^66^

Zhang et al. reported the synthesis of laccase‐Cu_3_(PO_4_)_2_ hNFs and application of UV‐Vis colorimetric sensor for epinephrine. In comparison with free laccase, laccase‐Cu_3_(PO_4_)_2_ hNFs showed quite high enzymatic activity and kinetic parameters due to the synergistic interaction between laccase and Cu^2+^ ions of Cu_3_(PO_4_)_2_. Epinephrine was able to be detected with good selectivity in a wide linear detection range (0.4–400 μg mL^−1^) and low detection limit (0.1 μg mL^−1^) using this strategy.^68^ Additionally, HRP‐Cu_3_(PO_4_)_2_ hNFs was confirmed to be a highly efficient UV–vis colorimetric sensor for the detection of H_2_O_2_. The results demonstrated that HRP‐Cu_3_(PO_4_)_2_ hNFs had rapid response, high sensitivity and good reusability for the detection of H_2_O_2_ compared to free enzyme assay.^31^, ^69^

In a study by Zhai et al. reported a colorimetric sensor array based on AChE hNFs for the detection of metal ions in water samples. Three types of hNFs (AChE‐Cu_3_(PO_4_)_2_ hNFs, AChE‐CaHPO_4_ hNFs and AChE‐Zn_3_(PO_4_)_2_ hNFs) were prepared to construct a chemosensor array. In the presence of metal ions, the catalytic activity of AChE was inhibited because of the reaction of metal ions with sulfur groups in AChE. The distinct binding affinities between AChE and metal ions could cause varying degrees of inhibition of AChE, leading to unique three‐signal fingerprint patterns. In this way, the identification and discrimination of different metal ions in water samples was carried out using principal component analysis (Figure 3B).^67^ Additionally, Jin et al. carried out the colorimetric and electrochemical detection for on site monitoring of OPs using acetylcholine esterase/choline oxidase‐Cu_3_(PO_4_)_2_ (AChE/ChO‐Cu_3_(PO_4_)_2_) hNFs. These hNFs had natural enzyme properties and enzyme‐like activity, and they were able to catalyze coloration of 3,3′5,5′‐tetramethylbenzidine (TMB) as a chromogenic substrate and amplify the electrical signal output at the end of the tandem catalysis reactions. An AChE/ChO‐Cu_3_(PO_4_)_2_ hNFs‐based biosensor could detect paraoxon as a model analyte at fg mL^−1^ level.^70^ These studies show that hNFs of the same enzyme can be used in the determination of various analytes using different techniques.

Colorimetric detection by naked eye requires discoloration of the sample or sufficient distinction of color development and it can not be applied to quantitative measurement.^65^, ^71^ Although traditional instrument‐based colorimetric detection have been widely used for the analysis of various analytes, this technique requires expensive and bulky instruments and professional operators.^65^, ^71^ In this context, smartphones have been very popular in recent years and have become suitable alternatives for different approaches for colorimetric analysis. Smartphone cameras can be used to capture images and the color quantification can be performed using a smartphone application without additional equipment. The advantages of using smartphone‐based colorimetric detection include (i) to obtain qualitative and quantitative results based on interactions with target‐specific agents, (ii) simple detection without any complex and bulk instrument, (iii) low cost, simple operation and easy portability, and (iv) suitable for point of care testing.^71^, ^72^, ^73^, ^74^, ^75^, ^76^, ^77^

**FIGURE 4:**
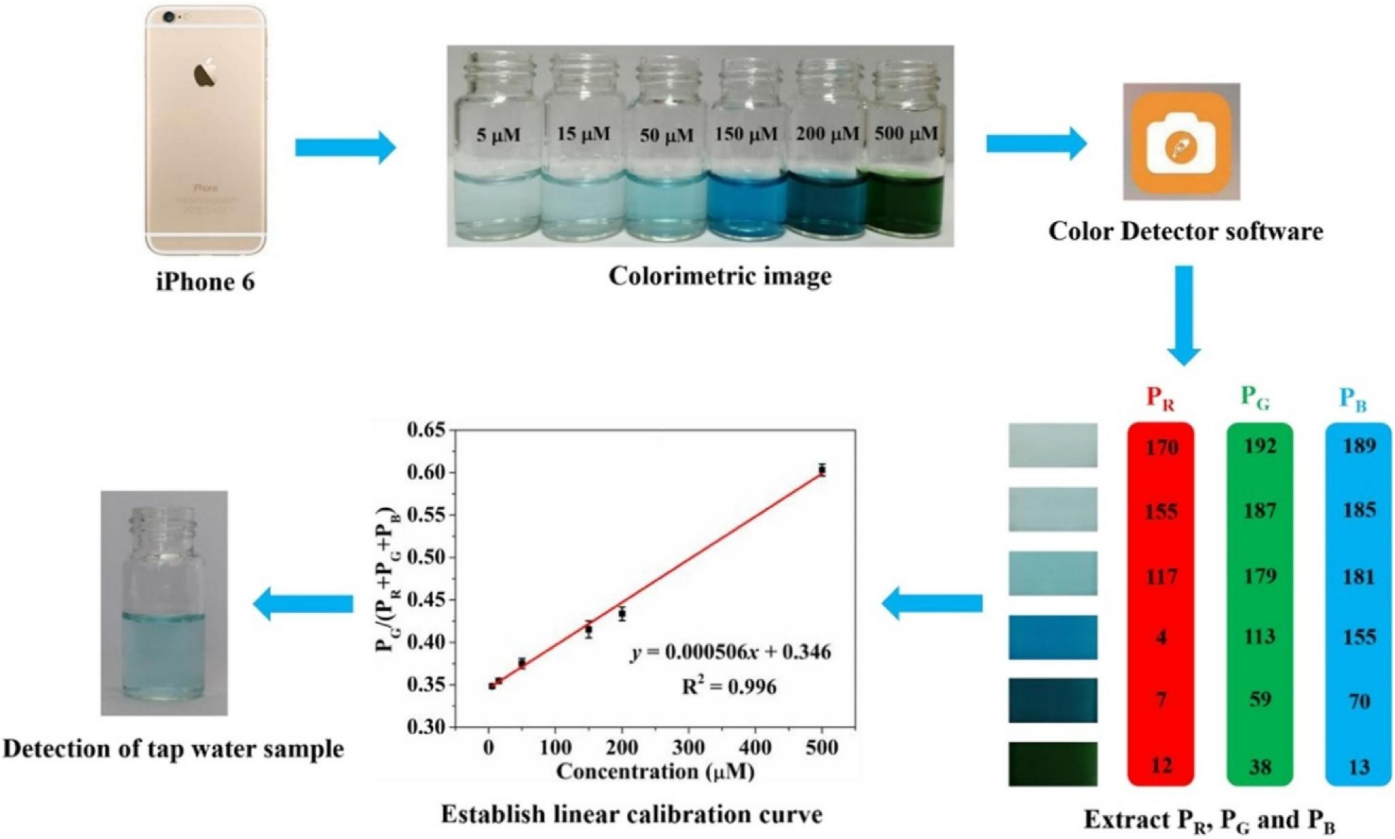
Smartphone‐assisted colorimetric platform using HRP‐Cu_3_(PO_4_)_2_ hybrid nanoflowers. Recently, the biosensors were developed on a paper substrate that provides a sensing platform for biological, environmental and clinical samples. Reproduced with permission.^78^ Copyright 2021, Elsevier.

In many studies, it has been shown that the colorimetric detection of H_2_O_2_ and glucose by using hNFs has been performed with high sensitivity due to the excellent catalytic activity of hNFs. In recent years, the colorimetric analysis of these analytes have been carried out via smartphone devices without using an optical device. Compared with the traditional device, smartphone devices give similar sensitivity and accuracy in the detection of H_2_O_2_ and glucose. For example, as shown in Figure 4, Zhang et al. designed a smartphone‐based colorimetric biosensor with HRP‐Cu_3_(PO_4_)_2_ hNFs for the ultrasensitive detection of H_2_O_2_. In this biosensor, in the presence of H_2_O_2_, HRP‐Cu_3_(PO_4_)_2_ hNFs catalyzed the oxidation of TMB, leading to color change. Colorimetric analysis was performed using a Color Detector software obtained via mobile application marker. The developed biosensor not only showed good selectivity and excellent accuracy but also had a wide linear range (5–500 μM) and low detection limit of 0.5 μM, which demonstrates great potential for biochemical detection.^78^ In another study, the same researchers reported a smartphone‐based colorimetric platform for glucose detection. GOx‐Cu_3_(PO_4_)_2_ hNFs were synthesized for the sensitive detection of glucose. Because of the fact that the easy to operate colorimetric platform showed a wide detection range, high sensitivity and good anti‐interference capability, it can be considered as a Laboratory‐on‐a‐Smartphone system.^79^ These studies demonstrated that the colorimetric detection of H_2_O_2_ and glucose can be carried out using smartphones in a simple and fast way. They will be the pioneer for simple analysis of various biomolecules using different enzymes in hNFs in the near future.

**FIGURE 5:**
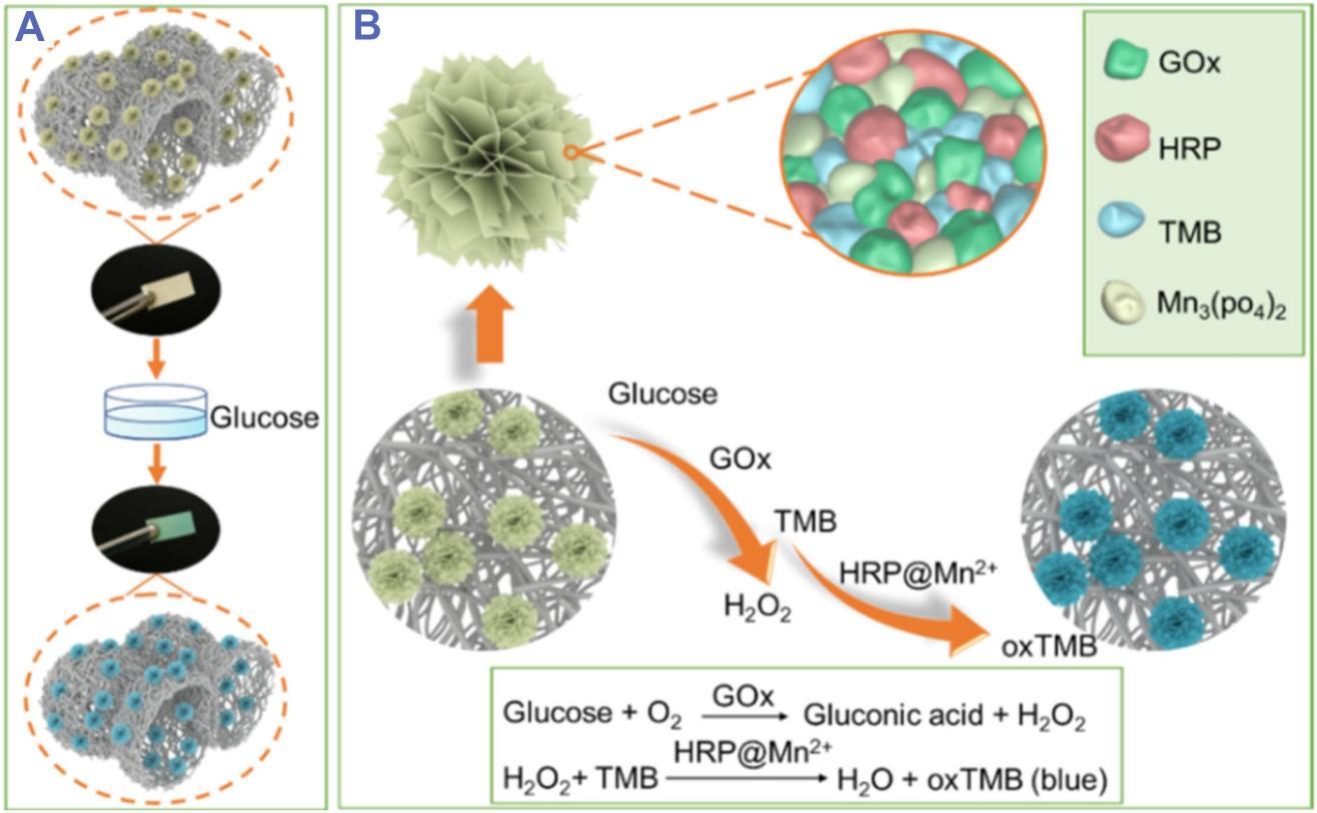
(A) Schematic illustration of the detection process, (B) The biosensing strategy for the detection of glucose using GOx–HRP–Cu_3_(PO_4_)_2_·3H_2_O nanofibrous strip. Reproduced with permission.^86^ Copyright 2022, Elsevier.

Paper‐based biosensors have drawn a broad attension due to their low cost, user‐friendly, portability and rapid response.^80^, ^81^, ^82^, ^83^ Especially, combining with smartphones provides improved analytical performance of biosensors. Furthermore, paper based biosensors fabricated with hNFs are an innovative method for the timely monitoring of different chemical compounds. In a study, Li et al. reported the colorimetric glucose detection on a microfluidic paper‐based analytical device (μPAD). In this study, GOx‐Mn_3_(PO_4_)_2_ hNFs were grown on cellulose paper by pipetting solution of MnSO_4_ and PBS containing a diluted enzyme. After glucose was catalyzed by GOx to form H_2_O_2_, Mn_3_(PO_4_)_2_, which showed the artificial peroxidase activity, reacted with H_2_O_2_ to turn into the colorless TMB to blue colored oxidized TMB. The blue color was captured by a smartphone camera. The natural and artificial enzyme immobilized μPAD performed colorimetric detection of glucose with detection limit of 0.01 M.^84^ Similarly, Avidad investigated μPAD for the detection of glucose using coupled GOx–HRP–Cu_3_(PO_4_)_2_·3H_2_O hNFs supported on a cellulose paper. This biosensor retained its activity up to 75 days under determined storage conditions.^85^ In another study, Luo et al. developed a porous nanofibrous test strip for the sensitive detection of glucose (Figure 5). TMB, GOx, HRP and Mn_3_(PO_4_)_2_ were immobilized onto hierarchical porous PVA‐co‐PE nanofiber strips. The prepared porous nanofibrous structure provided the growth of hNFs throughout the membrane due to its large surface area. The strip had a glucose detection limit of 0.14 mM and a linear detection range 0.25–10 mM. Furthermore, discernible color change by naked eye at low concentration of glucose and good anti‐interference performance were obtained from this developed strip.^86^

These colorimetric glucose sensors have convenient applicability for glucose detection in urine and blood serum samples. All of these possible applications of the biosensor will be the subject of the future work. Except for glucose detection, paper based biosensors based on hNFs have opened a new door for colorimetric analysis of different biomolecules. For example, Sun et al. fabricated phenylalanine ammonia lyase‐Ca_3_(PO_4_)_2_ hNFs (PAL hNFs) to develope paper‐based biosensor for the colorimetric detection of Phenylalanine (Phe) in urine samples. In this biosensor, PAL catalyzed Phe to form trans cinnamic acid (t‐CA), which can be oxidized by potassium permanganate, inducing the fade of potassium permanganate and color change on the paper substrate. This color change can be observed by the naked eye. In comparison with the other Phe detection methods, PAL‐Ca_3_(PO_4_)_2_ hNFs based paper based biosensors showed rapid response time (10 min). It revealed a detection limit of 60 μM with a linear range of 60–2400 μM. Because of urinary Phe value in PKU patients are below the 3000 μM, this biosensor can be effectively used for the detection of Phe.^87^ In another study, μPAD based on uricase/HRP‐Cu_3_(PO_4_)_2_ was prepared for the colorimetric detection of uric acid in whole blood. Using dual hNFs in μPAD enhanced the enzymatic activity and stability and the developed μPAD can afford costly effective colorimetric detection of uric acid. This sensing strategy may provide a highly efficient and robust platform for colorimetric analysis in biomedical applications.^88^

#### 3.2.1 Optical/colorimetric immunosensors based on hNFs

Enzyme‐linked immunosorbent assay (ELISA) is an immunoassay method combining specific interactions between antigens and enzyme‐linked antibodies. ELISA occurs on the solid substrate surface including microplate wells, paper materials, and magnetic beads. In the process of ELISA, an antigen is bound to its specific antibody attached to a solid substrate to form an antigen‐antibody complex. Then, the enzyme labeled antibody combines with antigen‐antibody complex, resulting in the formation of antibody‐antigen‐antibody complex. In each coating step, the substrate is washed with washing buffer to remove free enzyme‐labeled antibody. Finally, chromogenic reagents are added, which are catalyzed by enzymes to generate color variations. The intensity of color can be measured quantitatively to detect the concentration of the antigen.^89^, ^90^, ^91^, ^92^ ELISA is considered as the gold standard method for the detection of various analytes in all kinds of samples in applications ranging from food analysis to biotechnological and environmental disciplines.^90^, ^93^ Because the performance of ELISA depends on enzyme activity and enzyme loading capacity, the loss of enzyme activity in a short time under extreme pH and high temperature conditions causes the restriction of use of ELISA.^94^, ^95^ Recently, hNFs can be successfully introduced to ELISA, leading to improved enzyme activity and providing more functional sites for biochemical reactions owing to their high surface area.^96^, ^97^

**FIGURE 6:**
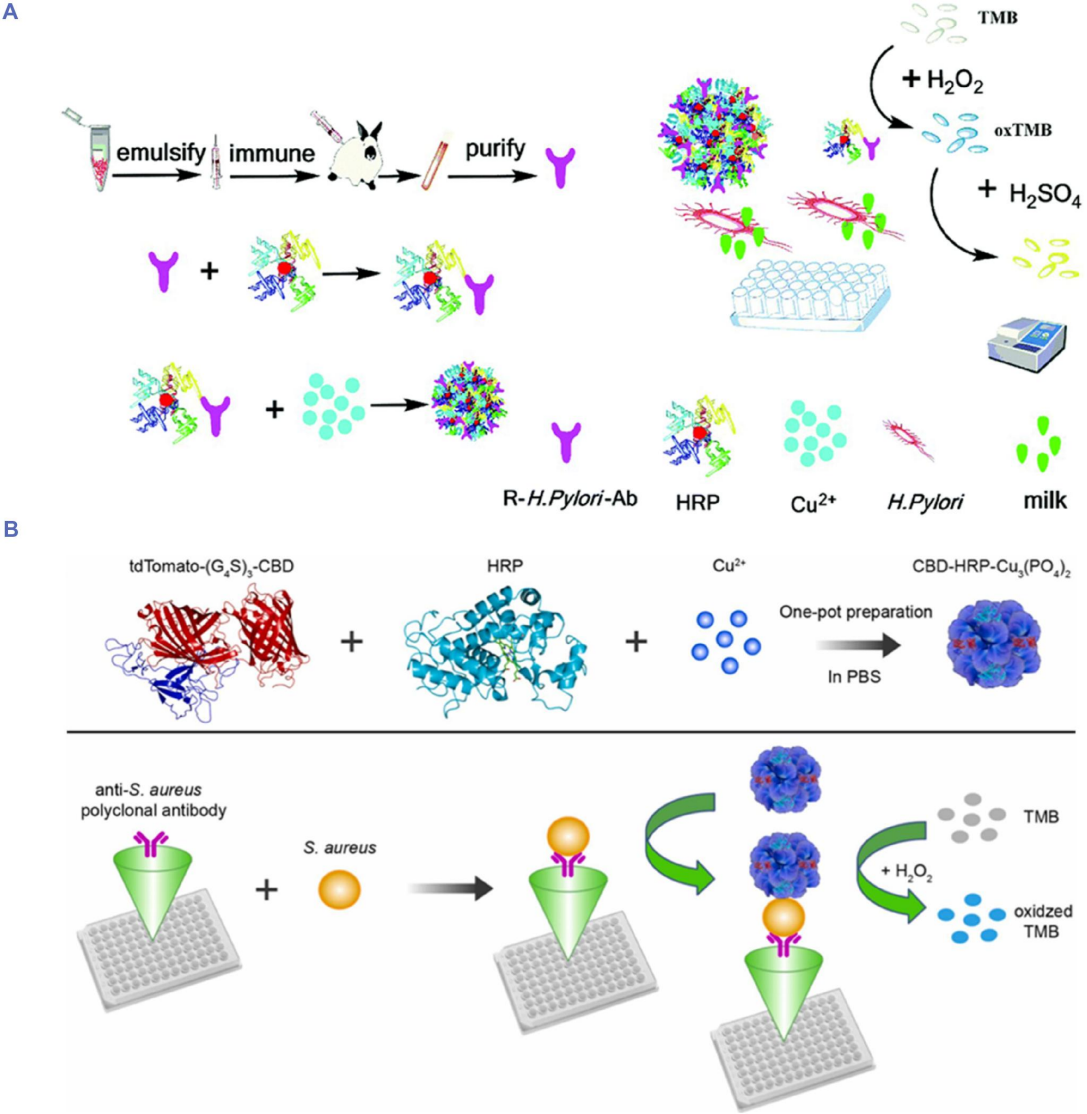
(A) Schematic illustration of the synthesis of hNFs and R‐HP‐Ab–HRP@Cu^2+^hNFs‐based immunosensor for the detection *Helicobacter pylori*. Reproduced with permission.^96^ Copyright 2021, Royal Society of Chemistry. (B) Schematic illustration of the synthesis of hNFs and principle of CBD‐HRP‐Cu_3_(PO_4_)_2_ hNFs based ELISA method for detection of *Staphylococcus aureus*. Reproduced with permission.^98^ Copyright 2022, Elsevier.

Wang et al. proposed a method based on indirect ELISA (iELISA) and hNFs including rabbit polyclonal antibody of *Helicobacter pylori* (HP) labeled with HRP (R‐HP‐Ab–HRP@Cu^2+^ hNFs) for sensitive detection of HP (Figure 6A). In the first step, HRP‐labeled R‐HP‐Ab was immobilized on the hNFs through natural complexation. In the presence of HP, R‐HP‐Ab–HRP@Cu^2+^ NFs recognized and captured the HP through specific antigen‐antibody interactions. The captured HP bound to R‐HP‐Ab labeled by HRP, resulting in catalyzing TMB in the presence of H_2_O_2_ to generate blue colored products. Finally, H_2_SO_4_ was added to terminate the reaction. The absorbance values at 450 nm were correlated with the concentration of HP in the range of 0–10^5^ CFU mL^−1^ in human artificial saliva. The generated NF‐based iELISA detection system exhibited outstanding performance, including 20 times higher sensitivity, acid‐base and storage stability compared with those of free enzyme‐labeled antibodies.^96^

Yin et al. reported an hNFs based ELISA method for the detection of *Staphylococcus aureus* (*S. aureus*) (Figure 6B). In this study, they used a cell binding domain (CBD) of the bacteriophage lysin PlyV12 derived from bacteriophage ϕ1, a fused protein tdTomato‐(G_4_S)_3_‐CBD, and a long flexible Gly4Ser linker (G_4_S)_3_ to construct three in one hNFs. Because of high specific binding ability of CBD with *S. aureus* and improved activity and stability of HRP, the CBD‐HRP‐Cu_3_(PO_4_)_2_ hNFs based ELISA method provided effective and sensitive detection of *S. aureus*, a detection limit as low as 6 CFU/mL in the range from 10^1^ to 10^6^ CFU/mL. CBD‐HRP‐Cu_3_(PO_4_)_2_ hNFs had several advantages including (i) simple preparation without involving covalent modification, (ii) hNFs provided higher detection sensitivity due to stronger adsorption capacity and large amount of CBD content, (iii) CBD‐HRP‐Cu_3_(PO_4_)_2_ hNFs had higher thermal resistance and storage stabilities than that of free HRP. Accordingly, this proposed ELISA method has an excellent potential in sensitive detection of *S. aureus* that causes human infectious diseases and food poisoning.^98^

In another study, Mao and Ye reported a magnetic chemiluminescence immunoassay platform on the basis of antibody‐enzyme CaHPO_4_hNFs using HRP as a signal amplifier for detection of *Salmonella enteritidis* (*S.enteritidis*). The platform consisted of immunomagnetic separation and a sandwich‐type immunoreaction. *S. enteritidis* was captured by streptavidin coated magnetic beads‐Ab (SMB‐Ab) and then *S. enteritidis* bound to the HRP‐Ab‐CaHPO_4_ hNFs. After magnetic separation, HRP catalyzed the oxidation of luminol in the presence of H_2_O_2_ and p‐iodophenol to produce a chemiluminescent signal. The intensity of the signal was proportional to the concentration of *S. enteritidis*. This developed platform exhibited high sensitivity, accuracy and excellent specificity for the detection of *S. enteritidis*.^99^

In addition to bacteria, the ELISA has become a powerful tool in the analysis of different molecules including disease biomarkers, proteins and pesticides. In recent years, scientists have shown that hNFs‐based ELISA has been used for high‐precision detection of different biomolecules. For instance, Su et al. reported ultrasensitive detection of Imidacloprid, a neonicotinoid pesticide, by using hNFs based ELISA. They prepared different hNFs (HRP@Ab_2_, Ab_2_@HRP, and HRP/Ab_2_) through controlled assembly of proteins and evaluated the effect of the distribution location of HRP and pesticide Ab on activity and stability. The performance of hNFs depended on the protein encapsulation sequence and HRP@Ab_2_ (Ab_2_ is on the exterior and HRP is in the interior of hNFs) demonstrated the best immunoassay activity because of its ultrahigh enzymatic activity and specific recognition capability. HRP@Ab_2_ hNFs exhibited excellent performance with a detection range from pg mL^−1^ to μg mL^−1^, providing broad range analysis of biomarkers in the biomedical applications.^100^

**FIGURE 7:**
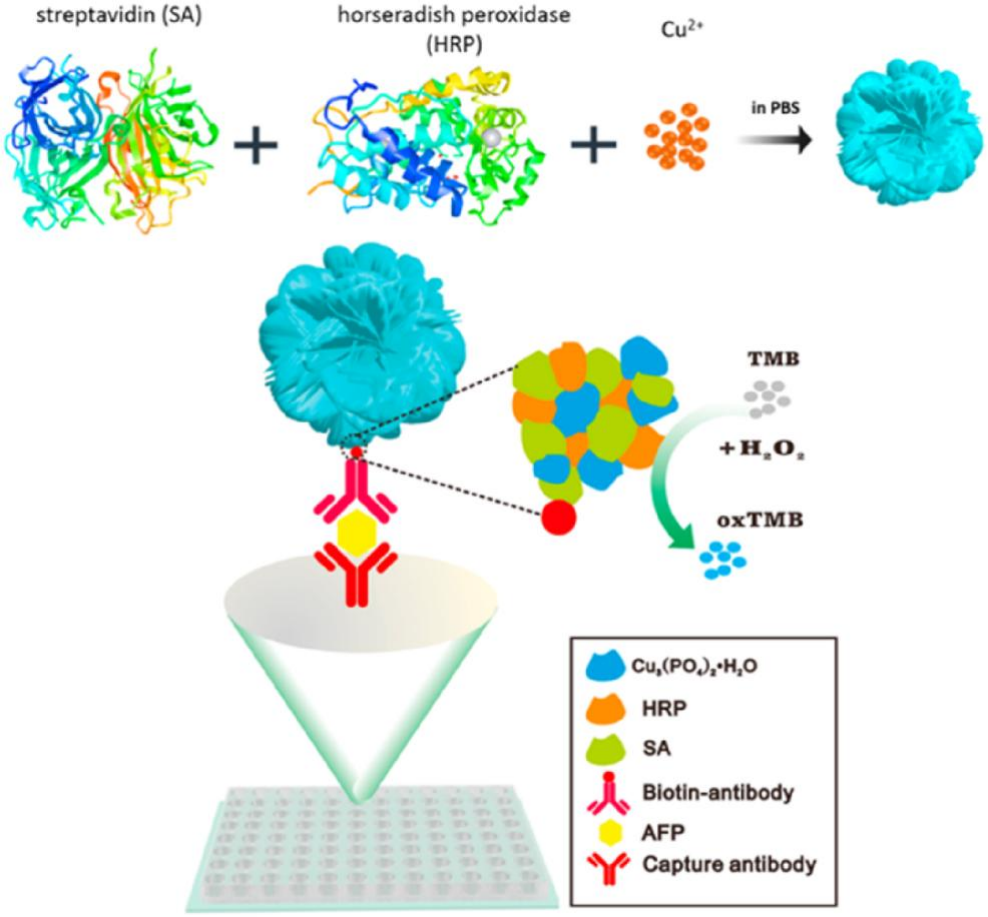
Illustration of the synthesis of SA‐HRP Cu_3_(PO_4_)_2_ and ELISA for AFP detection. Reproduced with permission.^101^ Copyright 2017, Elsevier. AFP, alpha‐fetoprotein.

Besides HRP, another type of enzyme can be used as signal amplification in ELISA. By altering the enzyme, different signal outputs can provide a more sensitive detection in hNFs based ELISA systems. Liu et al. synthesized dual functional hNFs, incorporating the functions of signal amplification and biological recognition for spectrometric detection of alpha‐fetoprotein (AFP; Figure 7). The synthesized streptavidin‐HRP Cu_3_(PO_4_)_2_ hNFs (SA‐HRP Cu_3_(PO_4_)_2_) captured biotinylated AFP antibody due to the specific interaction between SA and biotin. In the presence of AFP, HRP catalyzed the oxidation of TMB and generated a blue colored product. Using this strategy, the LOD of 78 pg mL^−1^ in the linear range of 0.1–50 ng mL^−1^ was achieved, which was more sensitive than commercial ELISA kits.^101^ On the other hand, the same researchers developed SA‐β‐galactosidase‐CaHPO_4_ hNFs (SA‐β‐Gal‐CaHPO_4_ hNFs)‐based fluorescent sensor. β‐Gal used as a signal amplification, catalyzed the fluorogenic substrate fluorescein di‐β‐D‐galactopyranoside into fluorescein giving fluorescence signal. SA‐β‐Gal‐CaHPO_4_ hNFs based fluorescent sensor could detect AFP in a linear range of 0.1–10 ng mL^−1^, reaching detection limit of 13 pg mL^−1^.^102^ These results show that biomolecules can be analyzed more sensitively by changing signal outputs using different enzymes.

## 4 CONCLUSION

In this review, the preparation of organic‐inorganic hNFs and their sensor application with various detection strategies are outlined. We reported that the HNFs are formed from organic and inorganic components. While the organic part can be enzymes and non‐enzyme molecules (aptamers, antibodies, standard organic or biomolecules and plant extact), the inorganic part is mostly composed Cu^2+^, Fe^2+^ and Zn^2+^ ions. The incorporation of enzymes with inorganic ions in the hNFs leads to enhancing the activity, stability and durability of enzymes due to the large surface area of hNFs, favorable enzyme conformation in nanoscaffolds and effective confinement enzyme molecules with mass transfer restrictions. The exceptional properties of hNFs make them highly promising for the development of biosensors due to their superior capture ability and excellent catalytic activity. Additionally, when the double enzymes are used in the hNF structure, the hNFs bring the two enzymes closer together, reducing the diffusion and decomposition of the substrate, which significantly enhances the reaction efficiency. Therefore, hNFs show great potential for biosensing applications.

Despite the rapid development of the hNFs in analyte detection, there are some problems to be faced in the future. In particular, hNFs‐based biosensors have remained at the research level and can not be applied in detection and diagnosis of real samples. In the near future, it may be possible to commercialize hNFs‐based biosensors by improving the analysis sensitivity and capture ability. Another important point is that the mechanisms involved in the synthesis of EHNFs are expected to be investigated more thoroughly, with a focus on exploring many details of the process. Also, more systematic investigation is required to explore the interaction between enzymes and metal ions in hNFs. This will provide some information for the design of hNFs with preserved catalytic activity and stability in the biosensor applications. The last point is that the hNFs should be synthesized with a narrow size distribution to reduce the inhomogeneous distribution of the signal tags, affecting the accuracy of the analysis. In summary, the development of new types of hNFs and their detailed mechanism will open up new opportunities in biosensor applications enabling the detection of different molecules with high accuracy and sensitivity and this will facilitate the commercialization of hNF‐based biosensors.

## AUTHOR CONTRIBUTIONS

Seyma Dadi and Ismail Ocsoy wrote the manuscript. Seyma Dadi prepared the figures. Seyma Dadi and Ismail Ocsoy revised the whole manuscript. Ismail Ocsoy supervised the manuscript.

## CONFLICT OF INTEREST STATEMENT

The authors declare that they have no known competing financial interests.

## ETHICS STATEMENT

In terms of ethics statement, we did not use any animal for experiment; hence, we did not need any ethics committee approval.

## Data Availability

The data that support the findings of this study are available on request from the corresponding author. The data are not publicly available due to privacy or ethical restrictions.
